# On the detection and attribution of gravity waves generated by the 20 March 2015 solar eclipse

**DOI:** 10.1098/rsta.2015.0222

**Published:** 2016-09-28

**Authors:** G. J. Marlton, P. D. Williams, K. A. Nicoll

**Affiliations:** Department of Meteorology, University of Reading, Reading, UK

**Keywords:** eclipse, gravity wave, pressure perturbation, radiosonde

## Abstract

Internal gravity waves are generated as adjustment radiation whenever a sudden change in forcing causes the atmosphere to depart from its large-scale balanced state. Such a forcing anomaly occurs during a solar eclipse, when the Moon’s shadow cools part of the Earth’s surface. The resulting atmospheric gravity waves are associated with pressure and temperature perturbations, which in principle are detectable both at the surface and aloft. In this study, surface pressure and temperature data from two UK sites at Reading and Lerwick are examined for eclipse-driven gravity wave perturbations during the 20 March 2015 solar eclipse over northwest Europe. Radiosonde wind data from the same two sites are also analysed using a moving parcel analysis method, to determine the periodicities of the waves aloft. On this occasion, the perturbations both at the surface and aloft are found not to be confidently attributable to eclipse-driven gravity waves. We conclude that the complex synoptic weather conditions over the UK at the time of this particular eclipse helped to mask any eclipse-driven gravity waves.

This article is part of the themed issue ‘Atmospheric effects of solar eclipses stimulated by the 2015 UK eclipse’.

## 1. Introduction

During a solar eclipse, the Moon passes between the Earth and the Sun, causing a shadow to move across the Earth. The shadow causes a transient cooling in the Earth’s atmosphere of up to a few degrees Celsius along the path of the umbra, which is the region of the Earth that experiences a total eclipse. Within the umbra, the solar disc becomes completely covered for a period of a few minutes, blocking all solar radiation with the exception of that emitted from the Sun’s corona. A smaller reduction in solar radiation and temperature can also be observed within the penumbra, which is the area where a partial eclipse is experienced.

An important consequence of the cooling effect of the Moon’s shadow during a solar eclipse was first hypothesized by Chimonas & Hines [1]. It was proposed that, as the Moon’s shadow moves at a supersonic speed through the Earth’s atmosphere, the associated cooling would cause internal gravity waves to propagate from the region of the shadow. Gravity waves are generated as adjustment radiation whenever a fluid departs from its large-scale balanced state [2–4]. Such a loss of balance will occur in the atmosphere when the horizontal temperature gradient suddenly changes due to the cooling during an eclipse. Simple analogies for this wave radiation process are the generation of surface gravity waves that form in the wake of a ship as it sails through the ocean, or the aerodynamic generation of acoustic waves that create sound as a plane flies through the air [5]. Chimonas [6] further developed the theory by predicting that the magnitude of the wave-induced pressure perturbations caused by an eclipse would be of magnitude 1 Pa at the Earth’s surface.

A number of investigators have found evidence of eclipse-driven pressure changes using sensitive micro-barometers. Measurements during the 7 March 1970 solar eclipse across the USA revealed pressure perturbations with a dominant period of 89 min, together with weaker perturbations with periods in the range from 15 to 57 min [7]. Similar measurements were made from Mauritania during the transit of the 30 June 1973 total solar eclipse across Africa. However, owing to large pressure changes associated with the local weather, eclipse-induced pressure perturbations could not reliably be calculated in this case [8]. During the 23 October 1976 total solar eclipse in southern Australia, pressure perturbations with a magnitude of 0.1–0.2 Pa were observed with a period of 23 min [9]. Waves with periods of 4 h were measured during the 11 June 1983 solar eclipse over southeast Asia by making multiple coordinated observations spanning a continental geographical range [10]. During the 11 August 1999 total solar eclipse in the UK, pressure perturbations with a period of 35 min were observed, and spectral techniques were used to demonstrate that this specific wave period was unique to the eclipse [11]. Most recently, during the 29 March 2006 solar eclipse in Greece, oscillations with periods of 20–50 min were observed in the ozone layer by examining the ozone photolysis rates calculated from total column ozone measurements [12]. The ozone oscillations were similar to oscillations observed in temperature and humidity at the surface, suggesting a possible link between the surface and mid-level atmosphere during the eclipse. Despite the encouraging findings of most of the above studies, it must be remembered that eclipse-induced waves are not always evident [13]. Care should be taken to separate perturbations that are eclipse-driven from those that are associated with normal meteorological influences. Table 1 shows a selection of eclipse pressure perturbation studies made in the last 50 years. The factors shown are the time of day, the distance that observations were made from the umbra, whether or not disturbed meteorological conditions were present, and whether or not a pressure perturbation was reliably found. Although the list may not be comprehensive or objective, it does outline how varying weather conditions can lead to a null result being reported.

**Table 1.** A list of previous eclipse perturbation experiments, with their observation distance, latitude of umbra nearest to observation, the presence of disturbed weather and indication as to whether a perturbation was observed.

eclipse	investigators	observation distance from umbra (km)	approximate local time of eclipse maximum nearest to observation	latitude of total eclipse nearest to observation	was disturbed weather present between the umbra and observation?	perturbations observed?
7 March 1970	Anderson *et al.* [7]	20	1200	30°N	yes	possible
30 June 1973	Jones & Bogart [14]	10	1130	2°N	no	no
30 June 1973	Anderson & Keefer [8]	40	1100	20°N	yes	no
11 May 1975	Jones [15]	2500	0630	69°N	yes	no
23 October 1976	Goodwin & Hobson [9]	500	1600	38°S	no	yes
9 March 1999	Jones [16]	5000	1200	57°N	yes	no
11 August 1999	Farges *et al.* [17]	20	1100	47°N	no	yes
11 August 1999	Aplin & Harrison [11]	100	1100	47°N	no	yes
29 March 2006	Zerefos *et al.* [12]	100	1300	36°N	no	possible

The present study examines temperature and pressure perturbations during the total solar eclipse that occurred over northwest Europe on 20 March 2015. The umbra tracked across the Atlantic and Arctic Oceans, where the only landmasses experiencing totality were the Faroe Islands, and Svalbard in the Arctic Circle. The penumbra, however, was experienced over most of northern Europe, with 85% totality across the British Isles and the Scandinavian Peninsula. Our study aims to examine whether gravity waves generated by the eclipse are detectable in surface temperature and pressure measurements from two sites in the UK: the Reading University Atmospheric Observatory (RUAO), which is located at 51.43° N, 0.93° W and experienced 85% totality, and the UK Met Office Lerwick Observatory, which is located at 60.13° N, 1.18° W on the Shetland Isles and experienced 97% totality. In addition, measurements above the surface are analysed from radiosonde balloons launched at the same two sites, to determine whether any eclipse-generated gravity waves are detectable in the upper atmosphere.

## 2. Methodology

### (a) Surface measurements

At the Reading Observatory, surface temperature was measured using a 100 Ω platinum resistance thermometer that was located inside a Stevenson screen. In addition, surface pressure was measured using a Druck precision barometer that is sensitive to 1 Pa and is identical to that used by Aplin & Harrison [11]. At the Lerwick Observatory, surface temperature was measured using an H&B PRT100 and surface pressure was measured using a Vaisala PTB220 sensor with an uncertainty of 5 Pa. Surface temperature and pressure measurements from Reading and Lerwick were all averaged over durations of 1 min, to increase the signal-to-noise ratio. The data were truncated to examine the 3 h period centred on the time of maximum occlusion, which was approximately 0930 UTC. This time window was chosen based on previous experiments, which have identified that the majority of the relevant wave perturbations are recorded between 15 min and 1 h from the eclipse maximum [9,11].

To examine rapid fluctuations in the surface measurements, both pressure and temperature time series were detrended using a smoothing spline to remove any relatively slow background variability. To minimize tapering effects around the edges of the selected time window, the smoothing spline was fitted to 6 h of data surrounding the eclipse and was then truncated down to 3 h. The residual data were then smoothed using a 10-point moving Savitzky–Golay method similar to that used by Zerefos *et al*. [12]. To examine periodicities in the residual data, Lomb periodograms [18] were taken, which tolerate missing data points and produce statistical significance values for each peak in the periodogram.

As discussed in §1, there may be other meteorological sources of gravity waves present. To attempt to determine whether any of the pressure perturbations detected are eclipse-driven, one can examine the wave propagation direction and speed to see whether any gravity waves are propagating away from the umbra. Information about the wave direction and speed can be calculated from the difference in the wind vectors between minimum and maximum pressure perturbations [19].

### (b) Upper atmosphere measurements

Given that the source of the gravity waves that are generated during an eclipse is in the upper atmosphere, and given that gravity waves propagate through the atmosphere, it may be possible to detect the gravity waves using radiosondes suspended from weather balloons. Such radiosondes have been used previously to investigate the properties of gravity waves in the troposphere and stratosphere using the temperature and horizontal wind component profiles [20,21].

Technological advances in the last 15 years have allowed radiosonde data to be archived at 1 s resolution. Therefore, vertical velocities can now be inferred from the radiosonde’s ascent speed and archived in addition to horizontal velocities. Vertical velocity variations in radiosonde profiles have previously been attributed to gravity waves [22] opening a complementary approach to observing gravity waves in the atmosphere.

**Figure 1.**
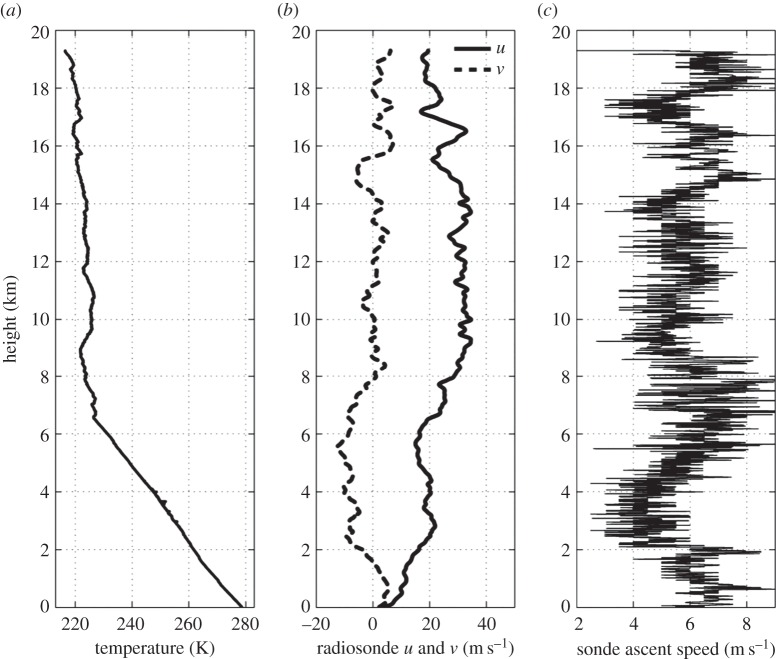
(*a*) Temperature, (*b*) *u* and *v* wind components and (*c*) vertical ascent speed from a Vaisala RS92 radiosonde ascent made from the Mesospheric, Stratospheric and Tropospheric Radar site at Aberystwyth, Wales, UK, on 3 March 2015.

To give an example of our analysis methodology, figure 1 shows data from a previous radiosonde ascent that was made over mountainous terrain (not during an eclipse). The ascent velocity shows the presence of a mountain wave across the depth of the tropopause. Further gravity wave activity is evident in the lower stratosphere. The intrinsic angular frequency *ω* of a gravity wave [23] can be calculated using
2.1
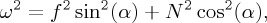
where *f* is the Coriolis parameter, *N* is the Brunt–Väisälä frequency and *α* is given by
2.2
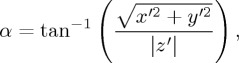
where *x*′, *y*′ and *z*′ are the three-dimensional displacements of an air parcel that is moving under the influence of the gravity wave. To a first approximation, the radiosonde can be considered to move as a passive air parcel, to which the following parcel displacement method analysis can be applied. To calculate the displacement perturbations from the radiosonde, the ascent speed and horizontal wind components are first smoothed to remove high-frequency instrumental noise. For this study, data spanning heights of 13–17 km are selected, as these were the heights at which maximum obscuration of the solar disc was observed by the radiosonde [24] and are above the nominal height of the tropopause in the mid-latitudes.

**Figure 2.**
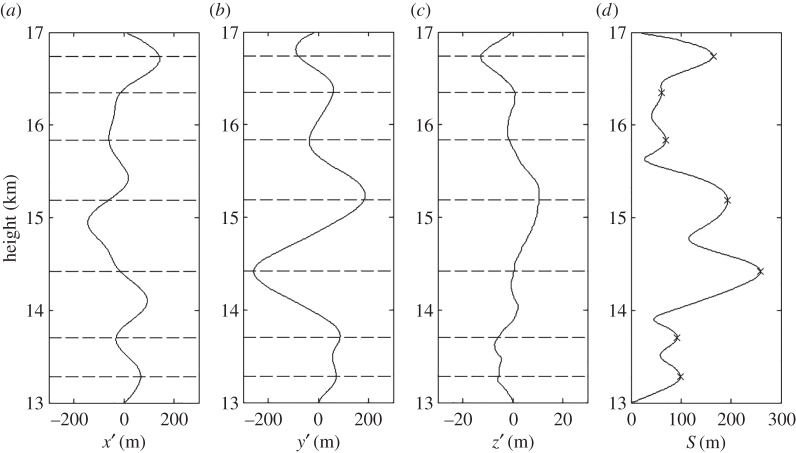
(*a*) Longitudinal displacement perturbation *x*′, (*b*) latitudinal displacement perturbation *y*′, (*c*) vertical displacement perturbation *z*′, and (*d*) magnitude of perturbations for all dimensions *S*. In (*d*), crosses mark the places where the analysis is applied. Dashed lines have been added to illustrate how peaks and troughs in at least two axes contribute to the maxima in *S*. These data are from a 13 to 17 km height window from a radiosonde ascent from Lerwick Observatory on 19 March 2015 at 0845 UTC.

A second-order polynomial was fitted to the *u*, *v* and *w* wind components to calculate the background velocities 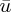, 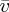 and 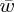. As a sensitivity test, several different polynomial fits were tested, but these did not significantly change the outcome, in agreement with [21]. The background velocities and true velocities were then integrated over the selected height window to yield the mean displacements 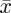, 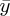 and 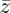. Because the horizontal displacement since launch is irrelevant in this analysis, *x* and *y* were integrated from the launch site and *z* was integrated from a height of 13 km. The mean displacements were then subtracted from the true displacements to yield the displacement perturbations, *x*′, *y*′ and *z*′. In order to select the points at which to perform the analysis, local maxima along the variable *S* were chosen, where
2.3
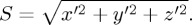
This choice was made because the contributing displacement perturbation magnitudes will then sum together to yield the largest detectable perturbation of the wave. If random fluctuations had been added together instead, they would have been less likely to yield a maximum. Figure 2 shows the selection of peaks and the contributing displacement perturbations for one of the ascents from Lerwick on the day before the eclipse. In this case, a large gravity wave is dominant between 14 and 15 km, with weaker gravity waves above and below it. To ensure that the perturbations shown in figure 2 are not simply noise, lines have been placed across the individual displacements for each dimension at maxima of *S*. In the case of no gravity waves being present, the alignment of troughs and peaks would be random across all three displacement dimensions. Figure 2 demonstrates that for all maxima along *S*, at least two peaks of the displacement dimensions are in phase. In cases where a third is not in agreement, it is often 90° out of phase, adding more confidence that the radiosonde is detecting a gravity wave.

Finally, *N* was calculated from the thermodynamic variables measured by the radiosonde over a 250 m height window using
2.4
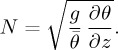
Here, *θ* is the potential temperature, the over-bar denotes an average over the height window, and *g* is the acceleration due to gravity. *N* is a measure of static stability, with *N*^2^<0 indicating that the air is hydrostatically unstable, thereby removing the restoring force that is required for gravity-wave propagation.

The characteristics of a monochromatic internal gravity wave can be inferred from a sounding by removing the background wind speeds and then plotting the horizontal velocity perturbations on a hodograph. For an ideal gravity wave, the hodograph would take the shape of an ellipse. By fitting an ellipse to an observational hodograph, the frequency of the gravity wave can be found. Specifically, the ratio of the major to minor axes of the fitted ellipse is proportional to *ω*/*f* [20]. This relationship allows the frequency of the gravity wave to be found from a hodograph if the Coriolis parameter is known. A disadvantage of this method is that one needs an almost complete ellipse to carry out the analysis. An advantage is that the wave propagation direction can be inferred from the orientation of the major axis of the ellipse, albeit with an 180° ambiguity [20]. Fortunately, this ambiguity can be resolved using wind and temperature data from the radiosonde. First, the velocity perturbation along the orientation of the major axis *U*^′^ is calculated. The temperature perturbation *T*′ is calculated in an identical method to the horizontal wind perturbations. For a gravity wave, *T*′ is ±90° out of phase with *U*′, hence by taking the vertical derivative of *T*′ the phase shift is removed and both quantities will have either the same or opposing signs. Hence, the sign of *η* can be used to deduce wave propagation, where
2.5
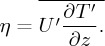
If the sign of *η* is positive (negative), then the wave propagates (anti) parallel to the positive bearing of the major ellipse axis [25], where the over-bar refers to spatial averaging over height. Wavelet transform techniques [26] have also been used to disentangle multiple coexisting gravity waves and allow information to be extracted about individual gravity waves. For the upper air data used here, ellipses are fitted only to complete ellipses observed in the hodographs. The thermal gradient is calculated over 100 m and *U*′ is sampled over the same height window. These are then averaged over the height range of the detected hodograph.

The moving parcel method is a useful tool as it enables the frequency of inertia-gravity waves to be inferred even if there is an incomplete or partial ellipse observed on the hodograph. However, as the moving parcel method uses the relationship between the vertical and horizontal components, it cannot be used to calculate wave propagation direction. The aim is to combine the strengths of these two methods to reliably gather as much information as possible about the inertia-gravity waves present in the atmosphere.

## 3. Results

**Figure 3.**
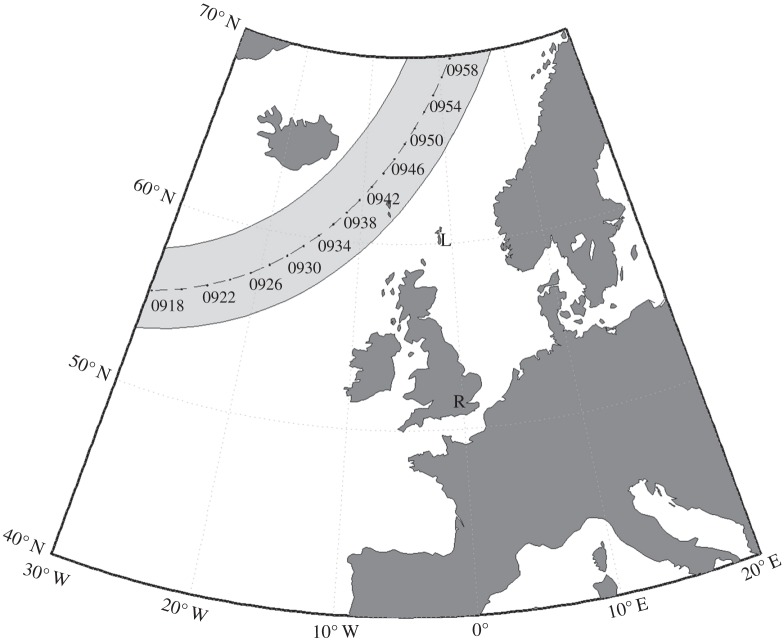
Path of the Moon’s umbra over northwestern Europe during the 20 March 2015 solar eclipse. Times of totality are marked along the umbra’s trajectory. The Lerwick and Reading observatories are marked with L and R, respectively.

The path of the 20 March 2015 solar eclipse transited across the North Atlantic Ocean as depicted in figure 3. The light grey shaded area indicates the path of the umbra, with the time of totality marked along the trajectory. Totality was experienced from 0908 UTC in the North Atlantic to 1020 UTC in the Arctic. At the Lerwick Observatory, 97% totality was experienced, with first contact at 0839 UTC and fourth contact at 1051 UTC. At RUAO, 88% totality was experienced, with first contact at 0824 UTC and fourth contact at 1040 UTC. In order to detect relatively weak perturbations in pressure and temperature caused by gravity waves, weather conditions would ideally be meteorologically quiet, because the passage of depressions and weather fronts can cause variations in temperature and pressure on hourly time scales. It is therefore imperative to begin by examining the synoptic weather conditions during the eclipse.

### (a) Synoptic conditions over the United Kingdom

**Figure 4.**
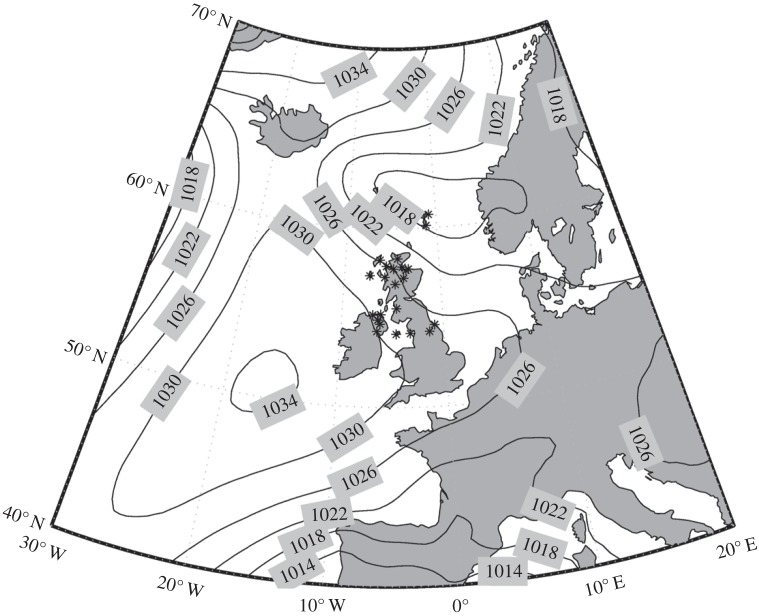
Surface pressure analysis over the UK at 0900 UTC on 20 March 2015 using ERA-Interim reanaylsis surface data. Black dots indicate automated weather stations where precipitation has been observed at the analysis time.

**Figure 5.**
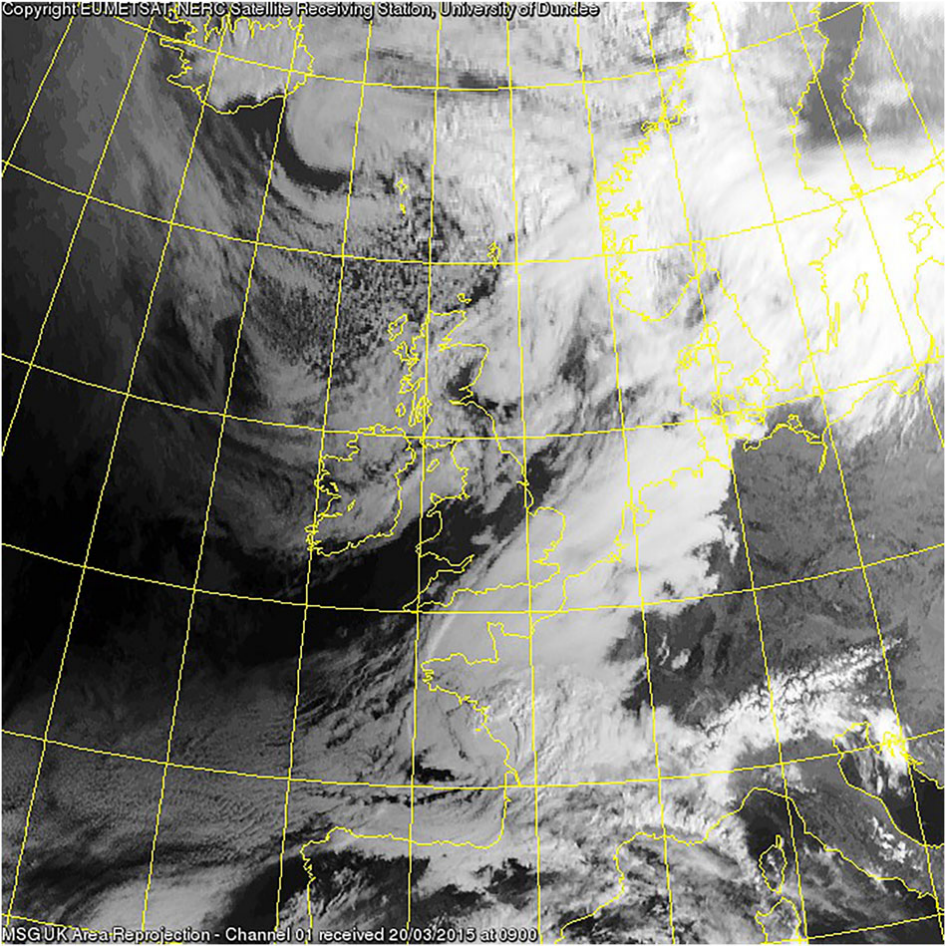
Visible image from the Meteosat SEVIRI geostationary satellite taken at 0900 UTC over the UK on 20 March 2015. Courtesy of the NERC Satellite Receiving Station, Dundee University, Scotland. http://www.sat.dundee.ac.uk/.

The surface pressure chart (figure 4) from the ECMWF ERA-Interim reanalysis [27] shows a large area of high pressure to the west of the UK, extending eastwards over central and southern UK. To the north, a low-pressure system moves zonally during the eclipse. Asterisks depict precipitation observed by automated weather stations, indicating frontal activity associated with the low-pressure system over northern England and Scotland during the course of the eclipse. Figure 5 shows the large amount of cloud associated with the system present over the northern UK. To the north-west of Scotland, cumulus rain showers can be seen, which are associated with the passing of a cold front. Over southern and central UK, a large swathe of stratiform cloud is present, which is associated with high pressure at this time of year. Finally, the visible satellite image shows the Moon’s umbra beginning to cross the Atlantic Ocean. In summary, the more complicated meteorological situation over Lerwick suggests that eclipse-induced wave phenomena may be less likely to be detected than at Reading, despite Lerwick being closer to totality.

### (b) Surface measurements at Reading

**Figure 6.**
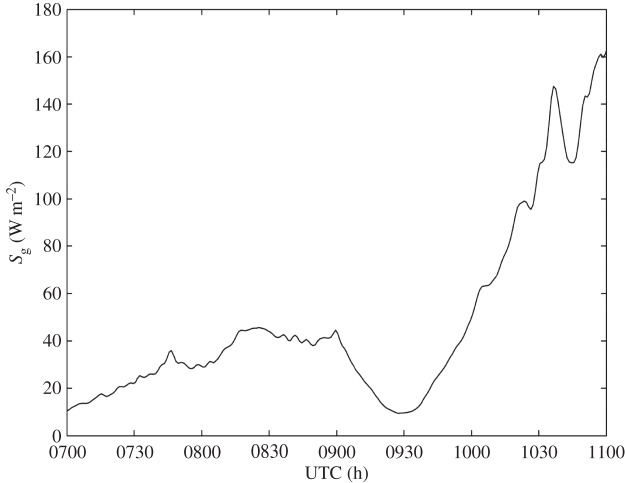
Global solar radiation, *S*_g_, observed using a Kipp and Zonen CM11 pyranometer at RUAO between 0700 and 1100 UTC on 20 March 2015.

**Figure 7.**
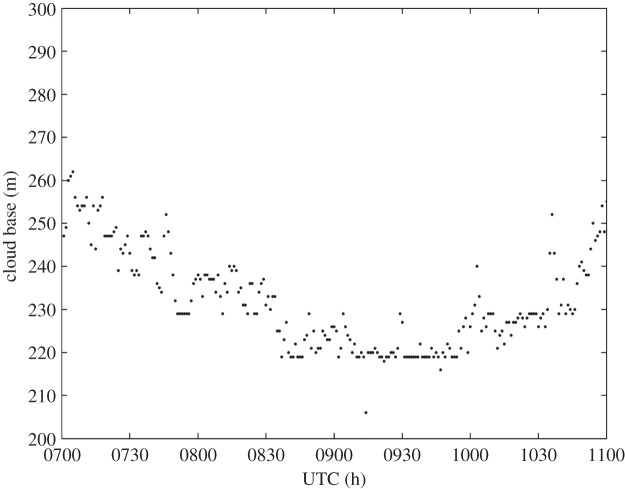
Cloud base recorded by Vaisala CL31 ceilometer at RUAO between 0700 and 1100 UTC on 20 March 2015.

For the entire duration of the solar eclipse, Reading was under the region of stratiform cloud that is shown in figure 5. This cloud is the cause of the low values of solar radiation that are shown in figure 6. Data from a Vaisala CL31 ceilometer in figure 7 show a cloud base height of around 250 m. Despite the presence of cloud cover, there is an obvious reduction of 20–30 W m^−2^ in solar radiation from 0900 to 1000 UTC during the eclipse, although this reduction would have been much larger in clear-sky conditions.

**Figure 8.**
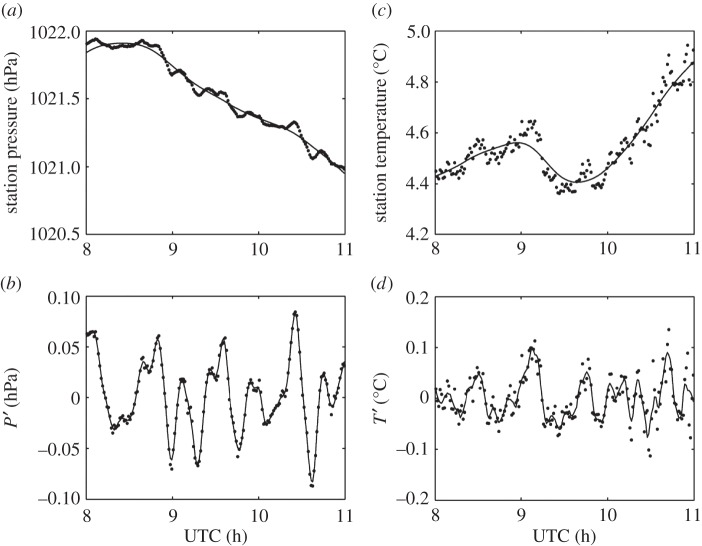
Surface measurements made from Reading during the eclipse on 20 March 2015. (*a*) Station pressure at 1 min resolution, where the smoothing spline is shown by the black line. (*b*) Detrended pressure data, where the black line indicates the Savitzky–Golay smoothing. (*c*) Dry bulb temperature, at 1 min resolution, where the smoothing spline is depicted by the black line. (*d*) Detrended temperature data, where the black line indicates the Savitzky–Golay smoothing.

Figure 8 shows time series of surface temperature and pressure at Reading between the hours of 0830 and 1030 UTC. Figure 8*a* shows that the surface pressure decreased by about 0.7 hPa during the eclipse. Although the timing of the start of the pressure decrease coincides with the decrease in solar radiation during the eclipse, the pressure drop is probably not eclipse-induced but rather caused by the ridge of high pressure becoming less dominant. The atmospheric cooling effect of the eclipse can be seen in figure 8*c*, which shows a 0.3°C surface cooling during the course of the eclipse. The cooling started at about 0900 UTC, when the solar radiation began to drop.

The corresponding detrended data points are shown in figure 8*b*,*d*. A Savitzky–Golay 10-point smoothing was applied, followed by a Lomb periodogram. Visual inspection indicates a 0.05°C drop in temperature between 0930 and 1000 UTC. This drop could be indicative of an eclipse-driven gravity wave, because a temperature change of this magnitude cannot be attributed to incoming solar radiation variability, which is minimal as shown in figures 6 and 7. Visual inspection indicates a wave period of approximately 30 min. In the pressure data, there are various wave forms in the residual data, and therefore a spectral analysis method is more appropriate.

**Figure 9.**
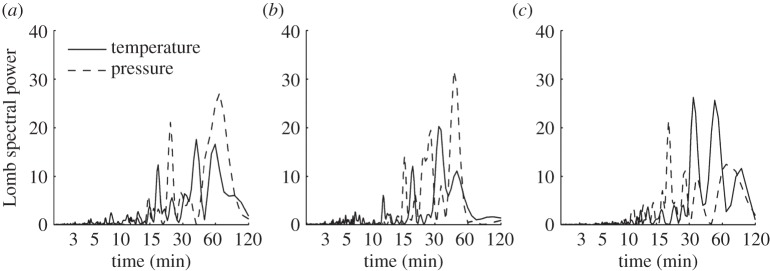
Lomb periodograms of the detrended temperature (solid) and pressure (dashed) for time series taken between 0800 UTC and 1100 UTC on (*a*) 19 March 2015, (*b*) 20 March 2015 and (*c*) 21 March 2015 at the RUAO.

**Figure 10.**
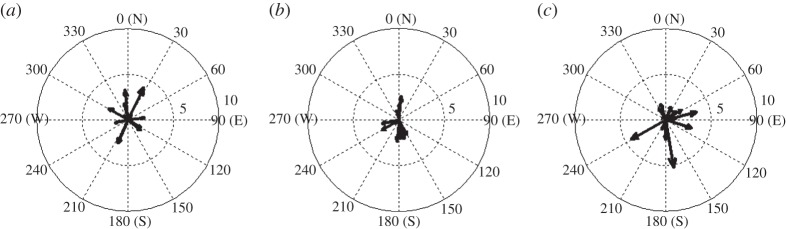
Compass plots showing wave speed (m s^−1^) and direction calculated using the difference of wind vectors at RUAO for 0800 to 1100 UTC on (*a*) 19 March 2015, (*b*) 20 March 2015 and (*c*) 21 March 2015.

The Lomb periodogram of the detrended surface temperature data in figure 9*b* shows a significant peak at a period of about 35 min, in good agreement with our initial visual inspection of the time series. The detrended surface pressure also shows a peak at periods of 45–50 min. However, in order to determine whether or not these peaks are attributable to the eclipse, we must assess the likelihood of such perturbations occurring on a non-eclipse day. Therefore, a similar analysis was performed on data recorded at the same time of day on the days both immediately preceding and following the eclipse. Figure 9*a*,*c* shows Lomb periodograms of the detrended surface temperature and pressure data from 19 and 21 March, respectively. It can be seen that on 19 March there are peaks in the temperature and pressure in the period range 45–60 min. On 21 March, there is a temperature peak in the period range 30–60 min, but for pressure the dominant peak occurs at a period of 20 min. In figure 10*b*, compass plots show the direction of wave propagation calculated from pressure and wind information assuming every perturbation in pressure is related to a wave. On the eclipse day, there are a large proportion of detected waves propagating to the south, which could be eclipse-driven waves propagating away from the umbra. However, when examining the days surrounding the eclipse in figure 10*a*,*c*, where waves propagate in multiple directions, there is little confidence in exclusively attributing the waves to the eclipse. Therefore, for the surface measurements made at Reading during the eclipse, we are unable to distinguish the temperature and pressure perturbations from those caused by other meteorological phenomena.

### (c) Surface measurements at Lerwick

**Figure 11.**
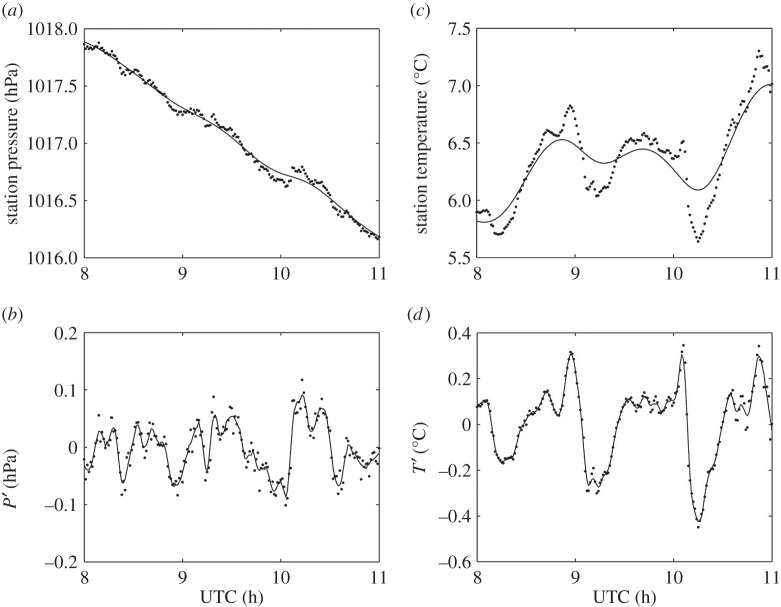
Same as figure 8, but for Lerwick.

A low-pressure system was passing over the Lerwick Observatory on 20 March (discussed in §3a), suggesting that it may be difficult to detect any eclipse-driven waves. Surface pressure and temperature from 0800 to 1100 UTC are shown in figure 11. From the surface temperature data, the onset of the solar eclipse is difficult to infer, because unlike in Reading (§3b) visual inspection indicates no obvious temperature anomaly. The surface pressure dropped by approximately 2 hPa during this period, which is attributable to the low-pressure system in the vicinity. In the residual pressure values shown in figure 11*b*, visual inspection indicates that a wave form could be present, but the reduced sensitivity of the barometer at Lerwick compared with the barometer at Reading makes the detection exercise more difficult.

**Figure 12.**
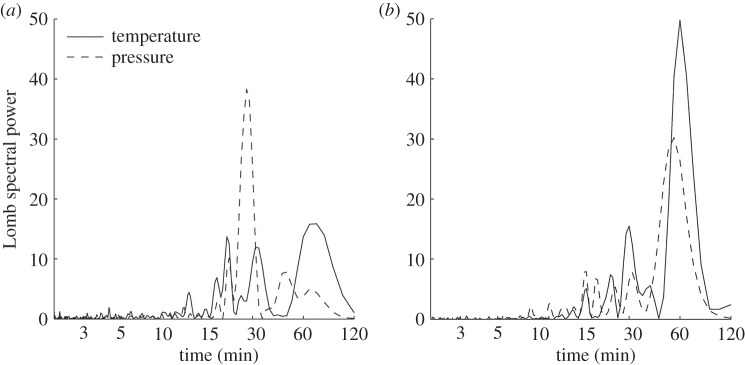
Lomb periodograms of the detrended temperature (solid) and pressure (dashed) for time series taken between 0800 and 1100 UTC at Lerwick Observatory on (*a*) 19 March 2015 and (*b*) 20 March 2015.

As in §3c, Lomb periodograms were constructed from the detrended surface pressure and temperature data collected at Lerwick for both 19 and 20 March, and are shown in figure 12*a*,*b*. The Lomb periodograms show temperature and pressure perturbations with periods of about 55–60 min during the eclipse. There is also a smaller peak at a period of approximately 30 min. For the non-eclipse day shown in figure 12*b*, there is a pressure perturbation peak at a period of approximately 30 min, indicating that there is no evidence to attribute the peak on eclipse day to the eclipse. There is a small peak at periods of about 60–90 min on 19 March, but the peak carries less statistical significance than the one detected on 20 March. Wave speed and direction were calculated for Lerwick between 0800 and 1100 UTC for both 19 and 20 March, as for Reading; however, for both days the derived values are random and do not show anything significant that would imply an eclipse-driven wave. The results are shown in the electronic supplementary material, figure S2.

### (d) Upper atmospheric measurements

**Figure 13.**
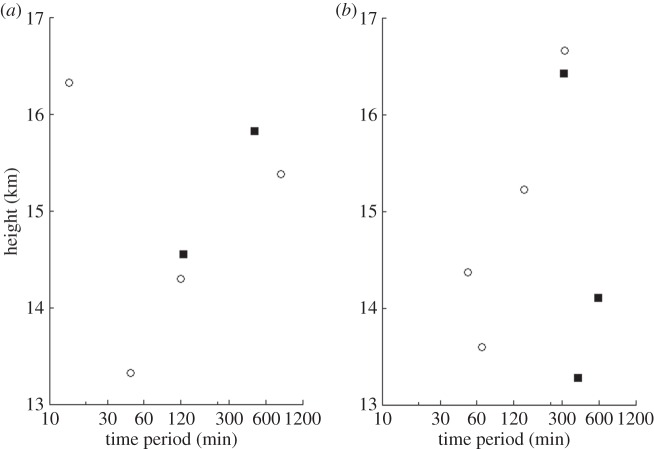
Periodicities of internal gravity-wave activity between 13 and 17 km for radiosonde launches using the moving parcel method (circles) and the hodographic method (filled squares) from Reading at (*a*) 0845 UTC on 19 March 2015 and (*b*) 0845 UTC on 20 March 2015.

Radiosonde launches made from the Lerwick and Reading observatories had reached the lower stratosphere when the eclipse was at its highest totality. The moving parcel analysis that was presented in §2b was performed on the data between heights of 13 and 17 km. At Reading, radiosondes were launched on the mornings of both 19 and 20 March. The period calculated from the moving parcel technique and hodographic technique is shown in figure 13*a*,*b*. As only two ascents were made, it is difficult to deduce whether the wave periods that were detected during the solar eclipse were caused by it. However, the periodicities that were deduced were typical of those of gravity waves in the lower stratosphere.

**Figure 14.**
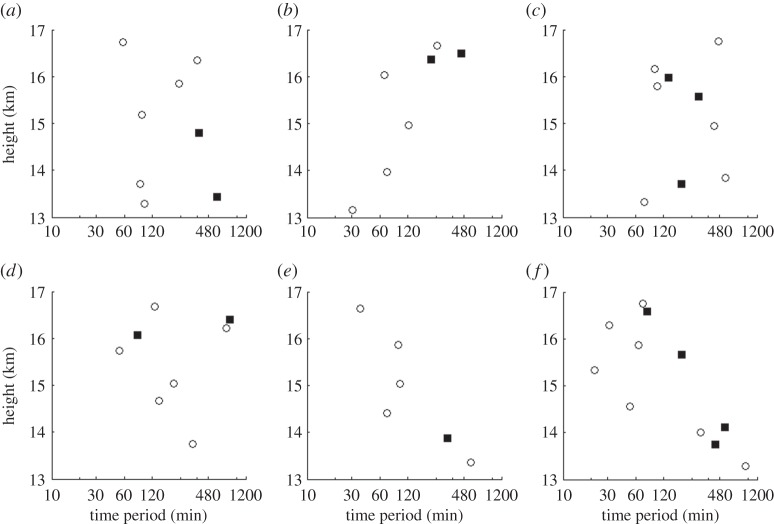
Periodicities of internal gravity-wave activity between 13 and 17 km using the moving parcel method (circles) and the hodographic method (filled squares) for launches at: (*a*) 0852 UTC on 19 March 2015, (*b*) 1500 UTC on 19 March 2015, (*c*) 2300 UTC on 19 March 2015, (*d*) 0854 UTC on 20 March 2015, (*e*) 1100 UTC on 20 March 2015 and (*f*) 1500 UTC on 20 March 2015, all from Lerwick Observatory.

At Lerwick, six radiosondes were launched over a 48 h period, being a mix of research and operational launches. Figure 14*a*–*f* presents time series of the radiosondes launched and the time periods derived from the moving parcel and hodographic analysis. A range of time periods is detected, with the majority similar to those of internal gravity waves. There is no evidence to suggest that the solar eclipse generated any distinctly different gravity-wave activity. The variability in the time periods for each ascent could be attributable simply to synoptic conditions and their interactions with the tropopause. During the ascent for the eclipse, only two complete ellipses were observed in the hodographs. Coupled with the large variability in propagation direction observed over the 48 h period, it is impossible to deduce whether the eclipse generated any gravity waves that were propagating away from the source of the eclipse. Propagation directions are shown in the electronic supplementary material, figures S3 and S4.

Although there is in general good agreement in the periodicities derived by the two techniques of moving parcel and hodographic analysis, figures 13*b* and 14*a* show large differences. In both cases, the moving parcel method appears to underestimate the period when compared to that of the hodograph, which may be attributed to errors in the moving parcel calculation method (discussed in equations (2.1)–(2.4)). Specifically, in the calculation of *S* (equation (2.2)), vertical and horizontal displacements may be phase shifted by 90° meaning that the full extent of the vertical or horizontal displacements may not be accounted for. Secondly, the hodographic analysis only uses horizontal velocity perturbations, therefore neglecting that the vertical velocity information may affect the periodicities detected from that using the moving parcel analysis. Finally, differences may also occur due to the reliance of the hodographic analysis on the selection of complete ellipses, of which more occur on some flights than others.

## 4. Discussion

### (a) Surface measurements

The results presented in §3b,c do not allow us to attribute any of the oscillations in surface pressure and temperature unambiguously to internal gravity waves generated as adjustment radiation in response to the cooling effect of the solar eclipse. At Reading, pressure and temperature perturbations were detected with periodicities of 30–55 min, which is similar to the periodicities observed in previous studies. A lack of variability in the cloud base height and solar radiation during the eclipse do suggest that the perturbations are not associated with weather systems. Wave propagation directions show little evidence to support exclusive eclipse-driven gravity waves at Reading. There may have been an eclipse-driven wave present, but it is impossible to detect it among other perturbations in pressure and temperature.

In [9,11], spectral analyses were carried out at different times to see whether the detected perturbations were exclusive to the eclipse. A similar approach has been adopted here. We found that at Reading, similar perturbations were found on different days, leading to the conclusion that it would be difficult to distinguish eclipse-driven gravity waves from normal meteorological variability. A similar situation occurred at Lerwick, although the pressure and temperature perturbations are present in the eclipse day data and not in the other data. The large-scale synoptic and mesoscale conditions near Lerwick could have explained the pressure and temperature perturbations. For example, a passing rain shower is identified in the satellite image. The Lerwick Observatory is based on the eastern side of a mountainous island, meaning that a mountain wave could form as the airflow is forced upward over the island, and such a mountain wave would have similar periodicities to an eclipse-driven gravity wave.

### (b) Airborne measurements

In §3d, the moving parcel and the hodographic techniques that were used to infer the periodicities of gravity waves in the lower stratosphere give time periods similar to those expected from the literature [23]. However, this technique did not detect gravity-wave periodicities during the eclipse that were significantly different from those detected during non-eclipse ascents. Furthermore, the hodographic direction technique did not resolve any eclipse-driven waves propagating away from the umbra at either site.

Chimonas [6] theorized that the ozone layer would be the internal gravity-wave source, due to cooling from a reduction in ultraviolet radiation, although the author did not rule out other parts of the atmosphere being sensitive to a decrease in solar radiation. During the 2006 solar eclipse in Greece, Zerefos *et al*. [12] reported similar oscillations in the ozone layer and at the Earth’s surface. This similarity indicates that internal gravity waves are generated by the eclipse-induced cooling at both the surface and in the ozone layer, which are both parts of the atmosphere that are sensitive to solar irradiance. This feature also provides a possible explanation as to why eclipse-induced pressure perturbations were not detected at the surface sites in this study. At Reading, the magnitude of the solar radiation decrease during the eclipse was about 40 W m^−2^, which caused a small cooling of 0.3°C. When compared to the solar radiation change of 600 W m^−2^ recorded during the 1999 solar eclipse [11,12] and the temperature drop of approximately 3°C recorded during the 2006 solar eclipse in Greece, it is possible that during the 2015 eclipse there was insufficient cooling at the surface to cause such a bow wave. This theory can be extended to Lerwick, where the temperature and pressure perturbations at the surface were dominated by synoptic effects. Both the low-pressure systems at Lerwick and the blanket of low cloud over the southeast UK would count as disturbed weather.

**Figure 15.**
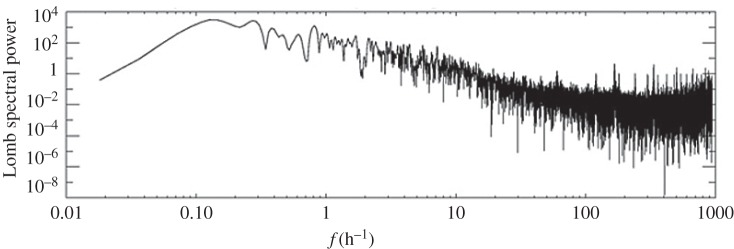
A Lomb periodogram of quadratically detrended data collected in the 14.5 h following the annular eclipse on 31 May 2003 (courtesy of Karen Aplin).

Other factors that could have affected the magnitude of the cooling are the time of day and year. During the August 1999 eclipse, the Sun’s zenith angle would have been lower and the eclipse was later in the day, causing larger cooling effects. In [7,9,12], pressure perturbations were observed at lower latitudes than the UK, which would also have appreciably reduced the solar zenith angle, further increasing the magnitude of the cooling effect. Sunrise is an extreme example of poor conditions in which to observe pressure perturbations during an eclipse. On 31 May 2003, an annular eclipse occurred over northern Scotland and the Arctic. The eclipse occurred near sunrise at 0400 UTC. A similar pressure sensor to the one that was used in the present study at Reading was deployed at Inverness. Figure 15 is a Lomb periodogram of detrended pressure over the 14 h following the annular eclipse. There are no statistically significant peaks in the spectrum, apart from that at 0.2 h^−1^, which is attributable to synoptic-scale weather.

Inadequate cooling near the surface could explain the absence of eclipse-induced pressure perturbations, but in the lower stratosphere there is less obscuration of solar radiation due to fewer clouds and less absorption and scattering. The lower stratosphere contains the ozone layer, which at high latitudes contains higher ozone concentrations at lower altitudes (15–20 km) [28]. This implies that a reduction in temperature could be experienced at this height during an eclipse. Muraleedharan *et al*. [29] showed temperature changes in the lower stratosphere; however, it is hard to attribute the changes due to the eclipse and not synoptic weather conditions which regulate tropopause height, and hence lower stratospheric temperatures. This would not negate internal gravity waves propagating downwards from higher up, whose amplitude may change in order to conserve energy. Given how the meteorological conditions in the troposphere can affect the tropopause, it could be likely that this could generate disturbances which could mask an eclipse-generated wave. It may also be a simple case that eclipse-driven internal waves are too subtle to be measured by the radiosonde’s GPS location system and pressure sensors.

## 5. Conclusion

This study has found little substantial evidence to suggest that pressure and temperature perturbations caused by eclipse-induced gravity waves could have been present over the UK during the 20 March 2015 solar eclipse. Contributions from the weather conditions, time of day and geographical location all obscured the interpretation of the perturbations, making them difficult to distinguish from perturbations caused by normal synoptic-scale influences. In particular, insufficient cooling due to cloud cover and the time of year could be a major factor as to why distinctive pressure perturbations were not detected. The results agree with other studies seeking eclipse-induced gravity waves, such as [8] and those discussed in [13], which have encountered similar problems due to disturbed weather conditions.

Further work might concentrate on making similar observations during a future solar eclipse. In particular, the solar eclipse of 21 August 2017 across the USA offers greater potential for detecting eclipse-induced gravity waves, because it will occur in the summer months at lower latitudes. In addition, a simple adjustment to the payload of the weather balloon could allow measurements to be made higher up in the atmosphere. This adjustment would improve the detection prospects for eclipse-induced internal gravity waves, because the density drop-off with increasing altitude causes wave amplitudes to grow quasi-exponentially with height in order to conserve kinetic energy. Finally, the moving parcel method used to derive frequencies could be further improved upon by a more comprehensive comparison with wind profiler observations of gravity waves with *in situ* measurements.

## Supplementary Material

Supplementary plots and diagrams for : On the detection and attribution of gravity waves generated by the 20 March 2015 solar eclipse

## Data accessibility

The meteorological data for each radiosonde ascent and the surface measurements made from Reading are available from the University of Reading’s Research Data Archive at http://researchdata.reading.ac.uk/id/eprint/5. Surface and operational radiosonde data from the Lerwick Observatory are available from the Met Office on request. ERA-Interim surface pressure data are available from the ECMWF. Station precipitation data are obtained from http://www.ogimet.com/synops.phtml.en.
