# Supplementary material for: On the detection and attribution of gravity waves generated by the 20 March 2015 solar eclipse
Source: Philos Trans A Math Phys Eng Sci. 2016 Sep 28;374(2077):20150222. doi: 10.1098/rsta.2015.0222 (PMC5004052; doi:10.1098/rsta.2015.0222)
Supplement: Supplementary plots and diagrams for : On the detection and attribution of gravity waves generated by the 20 March 2015 solar eclipse [file rsta20150222supp1.doc]

**Supplementary plots and diagrams for : On the detection and attribution of gravity waves generated by the 20 March 2015 solar eclipse**

In this paper methods are shown to calculate gravity wave propagation speed and direction from the pressure and wind vectors from surface data and through a hodographic analysis for radiosonde data. This supplementary material adds some detail to the calculation and results from both methods.

**Surface data**

A gravity wave as it passes a point causes perturbations in both pressure, P and wind speed and direction. By combining the pressure perturbation P’ and the wind vector information, wave direction and propagation speed can be calculated.

First at minimum P’ the wind vector is calculated then at maximum P’ a second wind vector is calculated. By subtracting the wind vector at P’max away from P’ min the wave direction and speed can be calculated. Figure 1 shows a vector diagram, denoting how the calculation is made (similar to that shown in [1]). The analysis is then carried out at each subsequent P’ minimum and maxuimum pairing along the time series


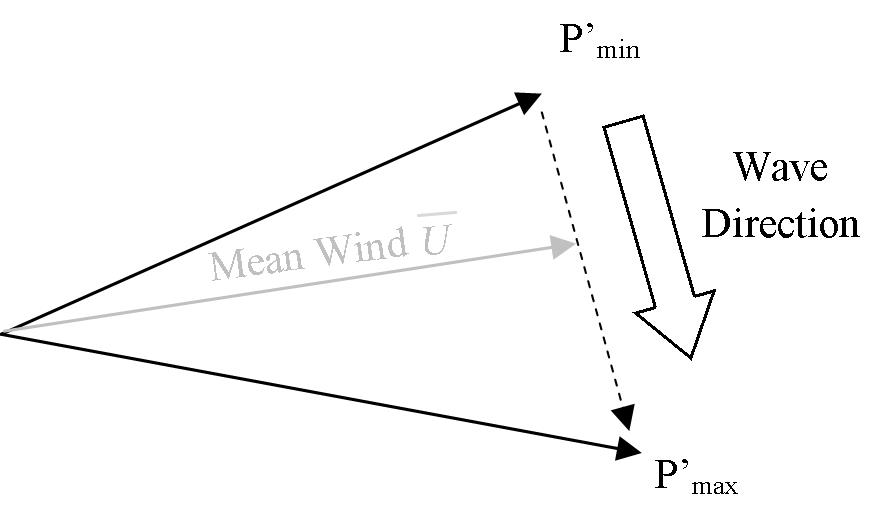


**Figure S1:** A vector diagram showing how wave direction and speed can be calculated by using the wind vectors taken from P’ minimum to P’ maximum.

The results of such an analysis using the wind and pressure data observed at the Lerwick Observatory are shown. The direction of the arrow infers wave propagation direction and the distance from the origin is the wave propagation speed.


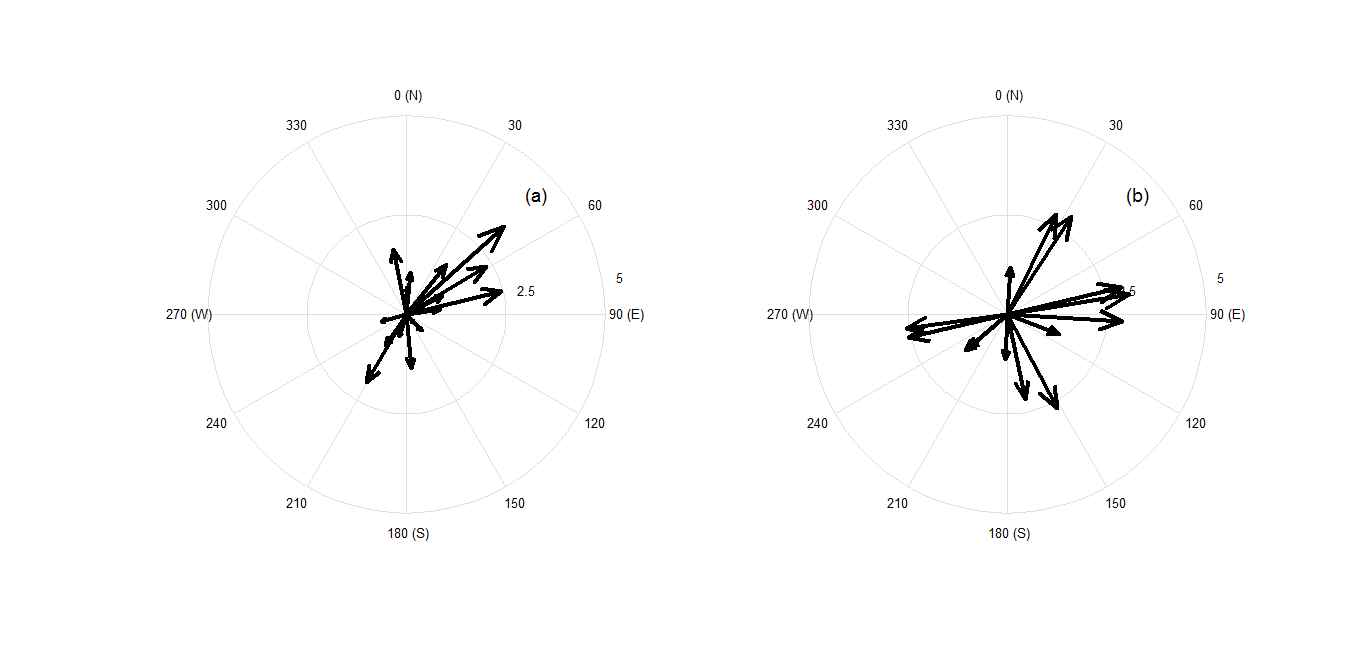


**Figure S2:** Wave direction and speed analysis based on a surface pressure perturbation and wind data between 0800UTC and 1100UTC on a) 19^th^ March 2015 and b) 20^th^ March 2015 from Lerwick Observatory

**Radiosonde data**

Figures S3 & S4 shown below are the results of using a hodographic analysis to infer information about the wave propagation direction (see section 3 of the main text for more information.) The method however is only able to detect the direction of propagation therefore in figures 3 & 4 the distance of the arrowhead from the origin does not reflect wave speed.


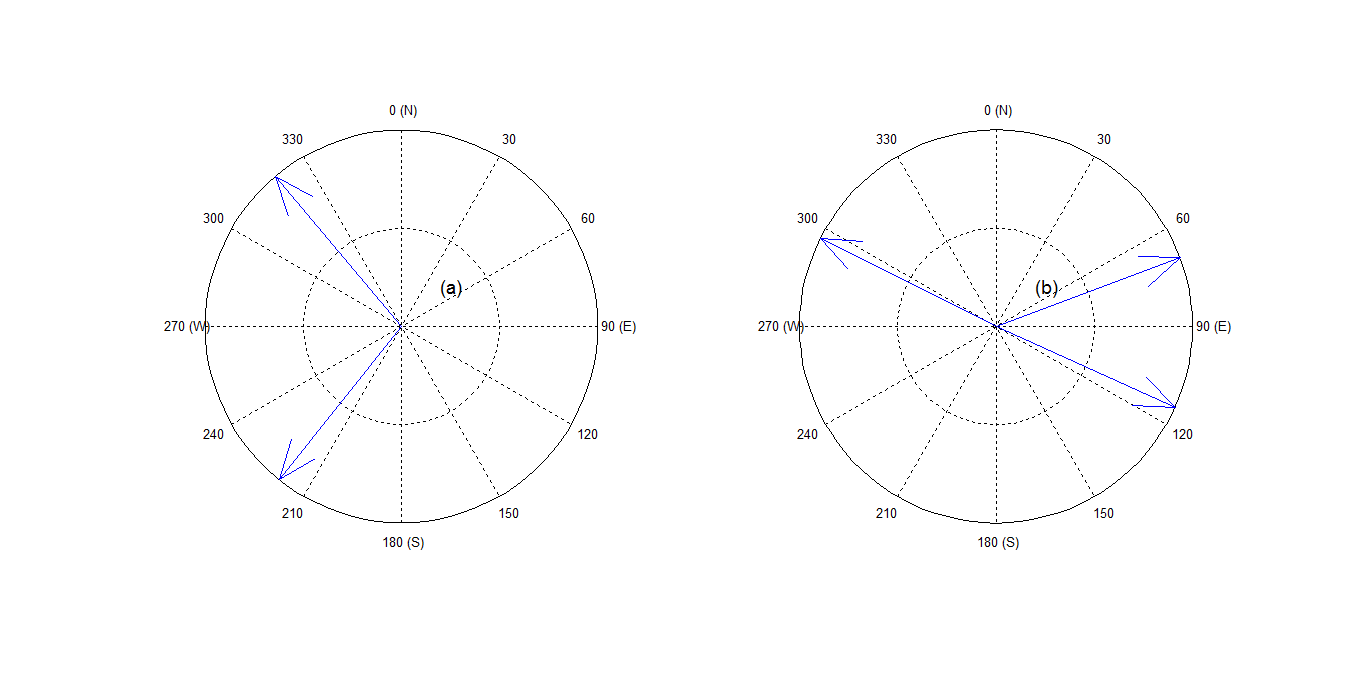


**Figure S3:** Wave propogation direction anaylsis from hodographs from radiosonde profiles made at Reading at 0845 on a) 19^th^ March 2015 & b) 20^th^ March 2015.


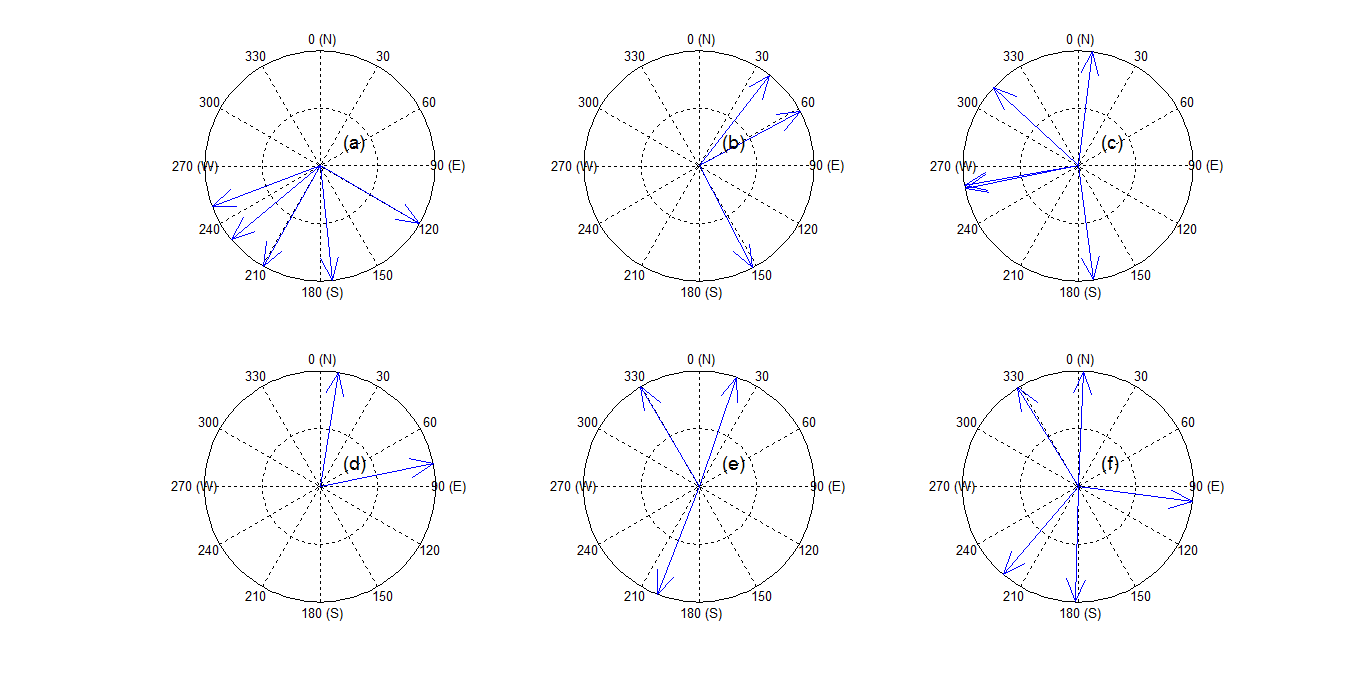


**Figure S4 :** Wave propogation direction anaylsis from hodographs from radiosonde profiles made at Lerwick at a) 0852UTC on19^th^ March 201, b) 1500 on 20^th^ March 2015, c) 2300 UTC on 19^th^ March 2015, d) 0854 UTC on 20^th^ March 2015, e) 1100 UTC on 20^th^ March 2015 and f) 1500 UTC on 20^th^ March 2015.

**References**

1. Gossard, E. E. and Hooke W.H., 1975, Waves in the atmosphere: Atmospheric infrasound and gravity waves-Their generation and propagation., Atm Sci, pp94, ISBN: 0-444-41196-8
